# Adolescent loneliness as a predictor of adult obesity: a longitudinal analysis from the HUNT study, Norway

**DOI:** 10.1186/s12889-025-23872-0

**Published:** 2025-08-13

**Authors:** Vegar Rangul, Marte Brennsaeter, Trine Tetlie Eik-Nes, Kirsti Kvaløy, Julie Friis

**Affiliations:** 1 HUNT Research Centre, Faculty of Medicine and Health Sciences, Department of Public Health and Nursing, Norwegian University of Science and Technology, NTNU, Forskningsveien 2, Levanger, NO-7600 Norway; 2 Levanger Hospital, Nord-Trøndelag Hospital Trust, Levanger, Norway; 3 Department of Community Medicine, Centre for Sami Health Research, UiT, The Arctic University of Norway, Tromsø, Norway; 4 Faculty and Medicine and Health Sciences, Department of Neuromedicine and Movement Science, Norwegian University of Science and Technology, NTNU, Trondheim, Norway

**Keywords:** Obesity, Loneliness, Adolescents, Epidemiology, HUNT, Prospective

## Abstract

**Background:**

Obesity and loneliness are growing public health concerns with potential long-term health implications. Although both issues have been extensively studied separately, the relationship between adolescent loneliness and adult obesity remains underexplored. This study aimed to investigate the associations between self-reported loneliness during adolescence and obesity in adulthood, analyze trends in loneliness and obesity among adolescents and young adults, and examine potential sex differences in these associations.

**Methods:**

This prospective study used data from The Trøndelag Health Study (HUNT) in Norway. The initial data collection focused on adolescents aged 13–19 years in 2006–2008 (Young-HUNT3), with a follow-up conducted in 2017–2019 (HUNT4) when participants were 23–31 years old; in total, 2,293 respondents (1,320 females and 973 males) were included. Loneliness was assessed using a single-item questionnaire, whereas obesity was assessed via body mass index (BMI) and waist circumference (WC). Multinomial logistic and linear regression models adjusted for covariates were used to analyze the associations between adolescent loneliness and adult obesity.

**Results:**

The prevalence of obesity increased significantly from adolescence to adulthood in both sexes, and the proportion of obese individuals increased from 4.0 to 16.6% in females and from 6.4 to 18.3% in males. Loneliness rates decreased in females but remained stable in males. Adolescents experiencing frequent loneliness showed a higher obesity risk in young adulthood than their less lonely peers. This relationship persisted after adjusting for confounding factors. Lonely adolescent males presented greater odds of BMI-defined obesity (OR 2.60, 95% CI: 1.12– 6.06) and greater BMI increases (1.97 kg/m², 95% CI: 0.38– 3.55) than females did (OR 1.82, 95% CI: 1.03– 3.22; BMI increase 1.16 kg/m², 95% CI: 0.01–2.31) later as adults.

**Conclusions:**

This study suggests that loneliness in adolescence may be a risk factor for BMI-defined obesity and increased WC in adulthood, with some variations in the strength of associations observed across sexes. The findings highlight the critical need to address loneliness as a public health concern and underscore the importance of a comprehensive approach to adolescent health, considering the long-term associations between social and emotional well-being on physical health outcomes.

**Supplementary Information:**

The online version contains supplementary material available at 10.1186/s12889-025-23872-0.

## Introduction

Obesity, defined by the World Health Organization (WHO) as a chronic and complex disease with multiple contributing factors, is a global public health challenge with significant implications for morbidity and mortality rates worldwide [1]. While commonly attributed to an imbalance between energy intake and expenditure, the underlying causes of excessive fat accumulation are multifaceted and involve biological, behavioral, psychological, social, and environmental factors. This complexity underscores the challenge of addressing the obesity epidemic on a global scale [2].

The prevalence of obesity has risen sharply over the past decade. Globally, adult obesity rates have more than doubled since 1990 [3], and the prevalence of abdominal obesity increased from 31.3 to 48.3% between 1985 and 2014 [4]. Similar trends have been shown in Norway, and the Tromsø Study reported an increase in the prevalence of obesity from 9.8 to 25.2% among men and 11.8–23.0% among women between 1994 and 2015 [5, 6]. The mean body mass index (BMI) rose by approximately one kg/m² per decade from 1984 to 2008 in Norway in both sexes [7]. Alarmingly, younger generations appear to be particularly affected by this increase in obesity. Longitudinal studies have shown a strong correlation between adolescent and adult BMI levels, and individuals born after 1970 present higher BMIs as young adults than earlier birth cohorts do [6, 8, 9].

Loneliness, the subjective experience of a gap between desired and actual social relationships [10], is a growing public health concern with potential adverse health implications. Unlike social isolation, loneliness is an emotional state that can reduce resilience to life stressors and negatively impact health outcomes [11]. Furthermore, people with obesity encounter higher levels of loneliness compared to those without obesity [12]. Among adolescents, loneliness has increased significantly in recent years both in Norway and globally [13, 14]. Several large-scale Norwegian population studies have investigated loneliness and associated health outcomes. The Tromsø Study, a longitudinal cohort initiated in 1974, targets adults aged 40 and above in Tromsø city and has provided important insights into how loneliness relates to both physical and mental health in older adults [15]. The Trøndelag Health Study (HUNT), among the largest in Norway, has conducted multiple survey waves since the 1980s. consistently examining the links between social factors like loneliness and a range of health indicators. Young-HUNT (YH), mainly a school-based surveys and a sub-study of HUNT, focuses on adolescents aged 13–19, collecting detailed mental and physical health data, including measures of loneliness. Findings from YH have shown that loneliness is associated with increased risk of mental distress, poorer self-rated health, and adverse health behaviors among adolescents. Notably, the proportion of lonely adolescents rose from 5.9% in the 1990 s to 10.2% in 2017–2019 in the YH Study [16].

Emerging evidence suggests that loneliness may contribute to adverse health outcomes such as mental health disorders, cardiovascular diseases, metabolic syndrome, and potentially obesity [17–21]. However, the relationship between adolescent loneliness and adult obesity remains unexplored. Addressing this gap could provide valuable insight into how early-life social experiences influence long-term physical health outcomes. Although gender-specific socialization and biological factors may influence variations in loneliness and obesity-related health outcomes [22], other sociodemographic variables such as age, socioeconomic status, educational attainment, ethnicity, and living arrangements also play a critical role [23]. For example, the link between loneliness and obesity is present across all age groups and is more distinct in individuals with lower socioeconomic position or less educational background. Additionally, factors such as experiences of discrimination, internalized weight stigma, and solitary living conditions may exacerbate susceptibility to both loneliness and obesity [24]. Identifying these differences is essential for tailoring interventions to address the unique needs for different demographic groups effectively.

This study seeks to explore the relationship between self-reported loneliness in adolescence and the likelihood of obesity in adulthood through three main objectives: (1) analyzing trends in loneliness and obesity among adolescents and young adults, (2) assessing whether adolescents who experience loneliness are at a greater risk of obesity as they transition into young adulthood, and (3) investigating potential sex differences in these relationships. Understanding these dynamics across various sociodemographic groups is essential for creating targeted interventions that address both the social and physical health issues associated with loneliness.

## Materials and methods

### Study population

This prospective study is part of the Trøndelag Health Study (HUNT) [25]. The HUNT Study comprises several waves (HUNT1 - HUNT4 and Young-HUNT), beginning in 1984. In each wave, all residents aged > 20 years and older (adolescents aged 13–19 years in Young-HUNT) in the county were invited to participate, making the sampling strategy essentially total population sampling within the defined geographic area. For this study, the initial data were collected from adolescents during the baseline period of Young-HUNT3 (YH3) in 2006–2008. The follow-up, focusing on young adults, was carried out between 2017 and 2019, the fourth wave of the HUNT Study (HUNT4). Adolescents aged 13–19 years living in Trøndelag County, Norway, were invited to participate in YH3 by completing a self-report questionnaire and were given a clinical examination during school hours. Eleven years later, in 2017–2019, the same group (now aged 23–32) was invited to HUNT4, completing a comprehensive health-related questionnaire at home and a clinical examination at a screening station.

**Fig. 1:**
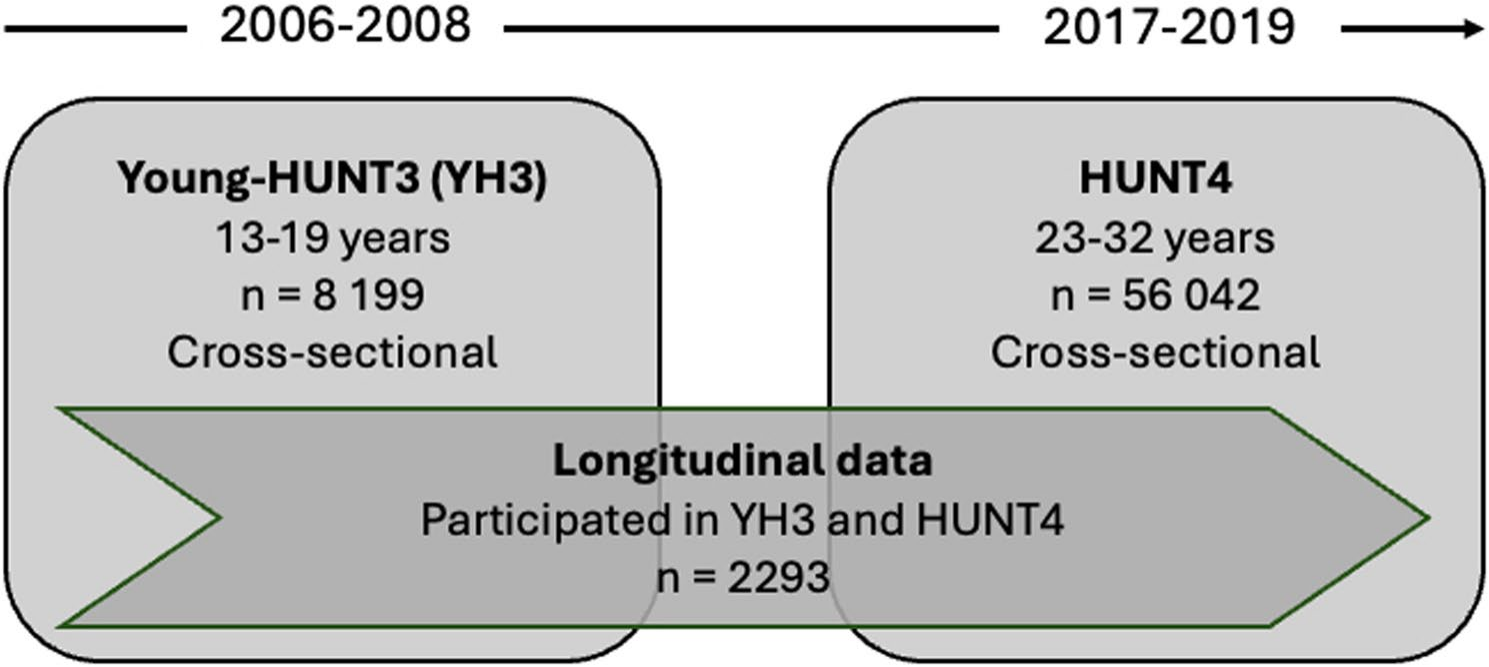
Flow chart of participants across the Young-HUNT3-HUNT4 surveys

In the YH3, 8,199 respondents participated (78.4% of those invited), and in HUNT4, 56,042 respondents participated (54% of those invited). This study included individuals who participated in both the YH3 and HUNT4 surveys, with a total of 2,293 respondents (1,320 females and 973 males). The flowchart in Fig. 1 illustrates the population in 2006–2008 and 2017–2019.

### Measurements

#### Obesity

The main outcome variables for body mass (obesity) were BMI and waist circumference (WC), assessed at two time points, in adolescence (YH3) and young adulthood (HUNT4), for the same individuals. BMI remains the most widely used metric for classifying overweight and obesity and is calculated as weight in kilograms divided by height in meters squared (kg/m²). Both outcomes in the adolescents and adults were assessed in a clinical examination via standardized methodology by specially trained nurses or technicians measuring weight to the nearest half kilogram with the participants wearing light clothes and no shoes, and height to the nearest centimeter [26]. BMI-based weight group characterizations in adolescence considering age and sex were calculated as provided by the guidelines from the International Obesity Task Force (IOTF), according to cut-off points by Cole [27]. As defined by the World Health Organization (WHO), obesity is defined as a BMI greater than or equal to 30 [28]. BMI has limitations as it does not reveal fat distribution or distinguish between fat and muscle mass. Centralized fat around the waist poses a greater health risk than fat distributed elsewhere [29], and WC is therefore considered a more precise measure of fat distribution and a stronger predictor of cardiovascular disease risk. In this study, the WHO cut-off for abdominal obesity for WC was used, corresponding to > 88 cm for women and > 102 cm for men [30]. WC and BMI in adults (HUNT4) were calculated from the body composition analysis through bioelectrical impedance analysis (BIA) (InBody 770, Cerritos, CA, USA). This system employs a tactile electrode system and multi-frequency currents (1 kHz–5 MHz) to segmentally measure impedance across five body regions (arms, legs, trunk). By analyzing resistance (flow opposition through intracellular/extracellular fluids) and reactance (cell membrane capacitance), the device estimates body composition metrics, including WC, through proprietary algorithms that correlate impedance-derived abdominal fat mass with anthropometric dimensions. The validity of BIA for WC estimation is supported by studies demonstrating strong correlations between BIA derived WC and manual measurements in adults [31]. These findings align with studies recognizing that BIA is a valid alternative to manual measurements for body composition determination thereby providing a more accurate measure than previous manual measurements such as WC for obesity categorization [32].

#### Loneliness

A single question to assess loneliness was used in the YH3survey asking participants: “Do you often feel lonely?“. A five-point response scale including “very often”, “often”, “sometimes”, “very rarely”, and “never” was used. For the main analysis, the responses were dichotomized into “rarely lonely” including the responses “never”, “very rarely” and “sometimes”; the other responses were classified as “lonely”. In a sensitivity analysis, the responses were divided into three categories: 1 = “very often and often”, 2 = “sometimes” and 3 = “very rarely and never”. In the HUNT4 Survey, the assessment of loneliness was also based on a single-item question, which asked participants how often they felt lonely. While a single-item question offers a simplified assessment of the complex and multidimensional nature of loneliness, Xu et al. [33] demonstrated that such single-item measures are strongly correlated with multi-item loneliness scales. This suggests that even brief assessments can effectively capture the core aspects of loneliness in population-based research.

#### Covariates

To guide our analysis and control for confounding factors, we created a directed acyclic graph (DAG) based on previous research and expert knowledge [34]. The DAG showed the expected relationships between our main exposure (loneliness), the outcomes (BMI and WC), and other related variables.

From the DAG, we identified confounders, such as age, sex, socioeconomic status (SES), number of close friends, mental distress, and self-rated health that might affect both loneliness and BMI. We included these confounders in our regression models to reduce bias. We also identified mediators, like physical activity and diet, which lie on the pathway between loneliness and BMI. These were not adjusted for, to avoid over-adjustment. Finally, we looked at whether age and sex might change the effect of loneliness on BMI by running separate analyses for different groups.

Education is frequently used as an indicator of SES in epidemiological studies [35]. Hence, educational attainment was assessed by the item “What plans do you have regarding continued studies” with the following five response categories: “None”, “College or university less than 4 years”, “College or university for 4 years or more”, “Vocational school or training” and “Don’t know”. College or university education of fewer than 4 years and for 4 years or more was considered “high education”, which was used as a proxy for high SES.

The participants were asked to rate their health with a single item question asking, “how is your health at the moment?“, with a four-point labeled scale. The responses were dichotomized as “not so good” including responses “poor” and “not very good”, and “good” including responses “good” and “very good”.

Mental distress was measured using the 5-item Hopkins Symptom Checklist (HSCL-5). The adolescents were asked if they had experienced each of the following during the last 14 days: “Been constantly afraid and anxious”, “Felt tense or uneasy”, “Felt hopelessness about the future”, “Felt dejected or sad”, and “Worried too much about various things”. Each item was answered on a four-point scale: 1 = “Not at all,” 2 = “A little,” 3 = “Quite a bit,” and 4 = “Very much.” To calculate the HSCL-5 score, the responses to the five items were summed and divided by the number of items answered, resulting in a mean score ranging from 1 to 4. A mean HSCL-score was calculated across the five items, using mean cut-off score of ≥ 2 as symptoms of mental distress, in line with established practice [36].

Dietary habits were assessed using a food frequency questionnaire. Respondents reported how often they consumed specific food and beverage items. For this study, we included the following dietary variables: fruit, vegetables, candy/chocolate/other sweets, and sugar-sweetened beverages (e.g., cola or soda). Each variable was dichotomized with responses categorized as 1 = daily intake and 0 = less than daily intake.

Physical activity levels were measured with the question: “Outside of school hours, how many days per week do you engage in sports or exercise to the point of becoming breathless and/or sweaty?” Responses were dichotomized into 1 = physically inactive (< 4 days per week) and 0 = physically active (≥ 4 days per week).

### Statistical analysis

Sex-stratified descriptive data are presented as percentages for categorical variables and means with standard deviations for continuous variables. Differences between baseline and follow-up were tested by McNemar’s tests and paired sample T-tests. The main analysis involved the construction of multinomial logistic regression models and multiple linear regression models to assess the association between loneliness and obesity, both analyses were sex-stratified. This approach compared crude (age-adjusted) and fully adjusted models. The analysis aimed to evaluate the relationship between loneliness and obesity while accounting for various confounding factors in different model specifications.

The main analyses were conducted via listwise deletion. To rigorously evaluate the robustness of our findings, we conducted three sensitivity analyses: Firstly, linear regression models were re-run using pairwise deletion to retain participants with partial data, thereby reducing potential bias from excluding incomplete cases. Secondly, linear and logistic regression analyses were repeated after excluding participants already classified with obesity in YH3, ensuring temporal precedence and minimizing reverse causality. Thirdly, analyses were repeated using tertile-based loneliness categories (low/moderate/high) instead of dichotomization, addressing potential measurement error in this complex construct. The IBM SPSS Statistics software, version 29, was used for the statistical analyses.

## Results

### Characteristics of the study population

The study sample comprised 2,293 participants from YH3 and HUNT4, including 1,320 females (57.6%) and 973 males (42.4%). At YH3 participation, the average age was 15.91 years (SD = 1.77), with ages ranging from 12.9 to 20.9 years. At follow-up at HUNT4, the average age was 26.74 years (SD = 1.88), with a range from 22.8 to 32.0 years.

The proportion who reported loneliness in YH3 was 8.9%, with prevalence being 10.7% among girls and 6.2% among boys. The prevalence of obesity was approximately twice as high among lonely adolescents than among non-lonely adolescents in both sexes. Specifically, 5.9% of lonely girls and 12.5% of lonely boys were classified as obese. Additionally, the proportion of individuals categorized as overweight was greater among those who were lonely than among those who reported that they were rarely lonely (Table 1).

**Table 1:** Baseline characteristics of participants according to categories of loneliness (*n* = 2 134; females *n* = 1 257, males *n* = 877)

	**Females**		**Males**	
	**Rarely lonely**	**Lonely**	**Rarely lonely**	**Lonely**
	% (n)	% (n)	% (n)	% (n)
BMI^*^				
Normal/underweight (≤ 24.9)	78.4 % (825)	69.7 % (83)	73.9 % (587)	56.3 % (27)
Overweight (25.0-29.9)	18.3 % (192)	24.4 % (29)	20.0 % (159)	31.3 % (15)
Obesity (≥ 30.0)	3.3 % (35)	5.9 % (7)	6.0 % (48)	12.5 % (6)
Mental distress				
Low (HSCL-5 < 2)	81.4 % (903)	23.1 % (31)	93.1 % (760)	42.6 % (23)
High (HSCL-5 ≥ 2)	18.6 % (206)	76.9 % (103)	6.9 % (56)	57.4 % (31)
Self-rated health				
Good	91.2 % (1011)	73.7 % (98)	92.8 % (760)	68.5 % (37)
Poor	8.8 % (98)	26.3 % (35)	7.2 % (59)	31.5 % (17)
Close friends				
Two or more friends	98.0 % (1097)	84.4 % (114)	96.7 % (795)	83.3 % (45)
One friend	1.8 % (20)	11.1 % (15)	2.4 % (20)	5.6 % (3)
No friends	0.2 % (2)	4.4 % (6)	0.9 % (7)	11.1 % (6)
Socioeconomic status				
Higher education	40.3 % (409)	49.2 % (58)	29.3 % (225)	21.6 % (11)
Vocational education	15.0 % (152)	8.5 % (10)	27.3 % (210)	31.4 % (16)
No plans/don’t know	44.7 % (453)	42.4 % (50)	43.4 % (333)	47.1 % (24)
Diet				
Fruit, daily intake	63.0 % (703)	43.3 % (58)	47.6 % (387)	44.4 % (24)
Vegetables, daily intake	50.9 % (565)	32.8 % (44)	44.4 % (357)	35.2 % (19)
Sugar beverages, daily intake	20.5 % (228)	23.1 % (31)	41.2 % (335)	43.4 % (23)
Sweets, daily intake	10.5 % (117)	18.7 % (25)	15.4 % (125)	20.4 % (11)
Physical activity				
Active (≥ 4 days/wk)	37.3 % (414)	23.7 % (31)	44.8 % (365)	30.2 % (16)
Inactive (<4 days/wk)	62.7 % (695)	76.3 % (100)	55.2 % (450)	69.8 % (37)
Smoking				
Not daily/never	93.5 % (901)	83.2 % (84)	95.9 % (680)	85.7 % (36)
Daily	6.5 % (63)	16.8 % (17)	4.1 % (29)	14.3 % (6)
Alcohol				
Less than every two weeks	79.2 % (743)	74.6 % (88)	75.4 % (503)	64.1 % (25)
Every two weeks or more	20.8 % (195)	25.4 % (30)	24.6 % (164)	35.9 % (14)

^*^ Age and gender adjustments by International Obesity Task Force (IOTF).

Notably, mental health distress was more prevalent among lonely participants of both sexes. Among those who were lonely, 76.9% of females and 57.4% of males presented symptoms of mental health distress, whereas 18.6% of females and 6.9% of males reported symptoms of mental distress in those who were rarely lonely. A greater proportion of lonely individuals compared to those not lonely reported poor self-perceived health and had few or no close friends.

Educational attainment varied by sex among lonely adolescents. A slightly greater proportion of lonely girls (49.2%) had higher education plans compared to the rarely lonely girls (40.3%). Conversely, fewer lonely boys (21.6%) had higher education plans compared to those who were rarely lonely (29.3%).

Dietary habits differed significantly according to loneliness status. Among individuals who reported loneliness, 43.3% of girls and 44.4% of boys consumed fruit daily, compared to 55.6% of girls and 58.2% of boys who reported to be rarely lonely. A similar pattern was observed for vegetable intake, with 32.8% of lonely girls and 35.2% of lonely boys reporting daily consumption, in contrast to 48.1% and 50.7% among their rarely lonely counterparts, respectively. Conversely, the prevalence of daily consumption of sweets was higher among lonely individuals (18.7% of girls and 20.4% of boys) than among those who were rarely lonely (12.3% of girls and 13.9% of boys). Daily intake of sugary drinks followed the same trend, with 23.1% of lonely girls and 43.4% of lonely boys reporting consumption, compared to 15.7% and 28.6% in the rarely lonely groups. Dietary disparities were more pronounced among girls. Furthermore, physical inactivity was more prevalent among lonely individuals than among those who rarely experienced loneliness, with this trend particularly evident among girls.

### BMI, WC, and loneliness from adolescence to adulthood

A significant increase in average BMI and the proportion of overweight and obese individuals among both females and males was found between the measurement points (Table 2). Among females, the proportion with general (BMI-based) obesity increased fourfold, from 4.0 to 16.6%, whereas among males, the proportion nearly tripled, from 6.4 to 18.3%. At both time points, obesity was more prevalent among males than females. The average WC and the proportion of individuals with abdominal obesity (based on WC) also increased from adolescence to adulthood in both sexes. In females, the proportion of individuals with abdominal obesity increased from 34.8 to 48.7%, whereas in males, it increased from 20.1 to 27.5%. Compared with males, females had a greater prevalence of abdominal obesity at both time points. More individuals were classified with abdominal obesity than with BMI-based obesity among both sexes in both YH3 and HUNT4. This discrepancy was most pronounced in females, with 4.0% having obesity and 34.8% having abdominal obesity in YH3 and 16.6% and 48.7%, respectively, in HUNT4. There was also a notable change in loneliness between the measurement periods in women. The proportion of lonely females was significantly lower in HUNT4 than in YH3. In males, the proportions of loneliness remained consistent across both measurement points.

**Table 2:** Prevalence trends in BMI, waist circumference and loneliness from YH3 to HUNT4

	**Females**			**Males**		
	**YH3**	**HUNT4**		**YH3**	**HUNT4**	
	**% (n)**	**% (n)**	p-trend ^a^	**% (n)**	**% (n)**	p-trend ^a^
BMI, mean (SD)	22.14 (3.71)	25.48 (5.27)	<0.001	22.14 (3.76)	26.25 (4.83)	<0.001
BMI^*^			<0.001			<0.001
Normal/underweight (≤ 24.9)	77.5 % (955)	55.1 % (697)		72.9 % (679)	44.7 % (406)	
Overweight (25.0-29.9)	18.6 % (229)	28.4 % (359)		20.6 % (192)	37.1 % (337)	
Obesity (≥ 30.0)	4.0 % (49)	16.6 % (210)		6.4 % (60)	18.3 % (166)	
Waist circumference (cm), mean (SD)	77.17 (10.53)	90.82 (14.62)	<0.001	79.64 (10.45)	95.16 (15.39)	<0.001
Abdominal obesity^*^			<0.001			<0.001
Normal	65.2 % (810)	51.3 % (640)		79.9 % (745)	72.5 % (652)	
Obesity	34.8 % (432)	48.7 % (608)		20.1 % (187)	27.5 % (247)	
Loneliness			<0.001			0.912
Rarely lonely	89.3 % (1122)	93.8 % (1230)		93.8 % (823)	93.9 % (906)	
Lonely	10.7 % (135)	6.3 % (82)		6.2 % (54)	6.1 % (59)	

Females: YH3 *n* = 1,257, HUNT4 *n* = 1,312; Males: YH3 *n* = 877, HUNT4 *n* = 965

^a^McNemar’s test for categorical variables and paired sample t-test for continuous variables

^*^ Age and gender adjustments by International Obesity Task Force (IOTF), for YH3 (95^th^ percentile for abdominal obesity)

**Fig. 2:**
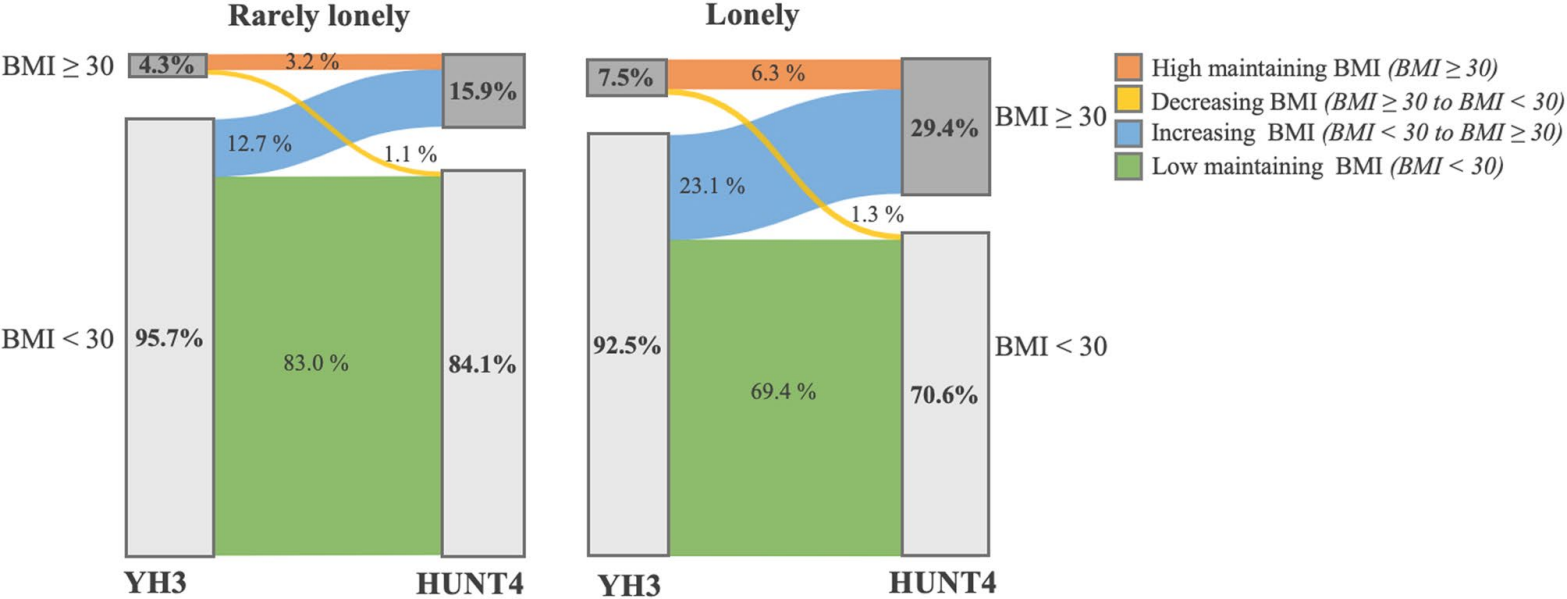
Variation in obesity rates (BMI ≥ 30) from adolescence (YH3) to young adulthood (HUNT4) among individuals who reported being rarely lonely versus lonely during adolescence

Figure 2 displays changes in obesity status within the same individuals across the two time points, illustrating within-person variation in obesity rates by loneliness groups. The prevalence of obesity increased more significantly in the lonely group (from 7.5–29.4%) than in the rarely lonely group (from 4.3–15.9%). The proportion of individuals who maintained obesity from YH3 to HUNT4 was greater among the lonely participants (6.3%) than among those who were rarely lonely (3.2%). The proportion of participants whose weight category was reduced to a BMI < 30 from baseline to follow-up was low and relatively similar across both loneliness groups. Notably, the proportion of adolescents who developed obesity in young adulthood between the measurement points was approximately twice as high in the lonely group (23.1%) than in the rarely lonely group (12.7%). These trends were consistent in both males and females.

### The impact of adolescent loneliness on the development of later obesity in young adulthood

The results from the multinomial logistic and linear regression models indicated that loneliness during adolescence was associated with an increased risk of obesity in young adulthood The estimated risk was slightly lower in the fully adjusted model, however the association between loneliness and obesity remained statistically significant in both sexes (females: OR 1.82, 95% CI: 1.03–3.22; males: OR 2.60, 95% CI: 1.12–6.06) (Table 3). Both regression models suggested an elevated risk of being categorized as overweight in young adulthood with association to loneliness in adolescence, although this relationship was not statistically significant for either sex.

**Table 3:** The association between loneliness in adolescence and BMI in adulthood (*N* = 2 017; females *n* = 1 253, males *n* = 764)

		Model 1 ^a^			Model 2 ^b^		
		OR overweight ^1^ (95% CI)	OR obesity ^1^ (95% CI)	Beta ^2^ (95% CI)	OR overweight ^1^ (95% CI)	OR obesity ^1^ (95% CI)	Beta ^2^ (95% CI)
Females	Rarely lonely	1.0	1.0	Ref.	1.0	1.0	Ref.
	Lonely	1.16 (0.75–1.79)	2.06^**^ (1.31–3.24)	1.62^**^ (0.66–2.58)	1.15 (0.67–1.96)	1.82^*^ (1.03–3.22)	1.16^*^ (0.01–2.31)
Males	Rarely lonely	1.0	1.0	Ref.	1.0	1.0	Ref.
	Lonely	1.06 (0.52–2.17)	2.86^**^ (1.43–5.71)	2.07^**^ (0.71–3.44)	1.34 (0.60-3.00)	2.60^*^ (1.12–6.06)	1.97^*^ (0.38–3.55)

^a^ Adjusted for age

^b^ Adjusted for age, socioeconomic status (SES), mental distress, close friends and self-rated health

^1^ Reference category: Normal weight/underweight

^2^ Unstandardized regression coefficients (B)

^*^ *p* < 0.05, ^**^ *p* < 0.01

Individuals who frequently experienced loneliness in their adolescence had, on average, a significantly higher BMI in young adulthood than those who were rarely lonely. This correlation persisted after adjustment. Specifically, females who were lonely during adolescence had an average BMI increase of 1.16 kg/m² (95% CI: 0.01–2.31) compared with those who were rarely lonely. In males, the difference was 1.97 kg/m² (95% CI: 0.38–3.55) (Table 3).

The sensitivity analysis, excluding initially participants with obesity, employing a three-category loneliness classification, revealed sex-specific outcomes (Table S1). For females, a slightly heightened risk of adult obesity, and a marginally increased average BMI was associated with adolescent loneliness, compared to the primary analysis. For males, adolescent loneliness was not significantly associated with risk of overweight in adulthood (OR 0.92, 95% CI: 0.43–1.98), while the odds of obesity were significantly increased in the age-adjusted model (OR 2.45, 95% CI: 1.10–5.50).

The inclusion of an “occasionally lonely” category in the analysis further supported the observed relationship (Table S2). Adolescents who reported occasional loneliness faced an elevated risk of adult overweight and obesity, along with higher average BMI, compared to those rarely experiencing loneliness. This trend was particularly pronounced in females.

Notably, individuals categorized as lonely exhibited an even more substantial risk of obesity and higher average BMI than observed in the main two-category analysis of loneliness. This suggests that the intensity of loneliness may correlate with the degree of weight-related outcomes in young adulthood.

Table 4 illustrates the relationship between loneliness during adolescence and WC in young adulthood. Both females and males who experienced loneliness in adolescence had larger WCs as young adults than did those who were rarely lonely in the age-adjusted model (Model 1). This association persisted only in males in the fully adjusted model (Model 2). Specifically, males who were lonely during adolescence had, on average, a 5.61 cm (95% CI: 0.54–10.67) greater WC in young adulthood than those who were rarely lonely.

**Table 4:** Multiple linear regression association between loneliness in adolescence and waist circumference in adulthood

		Model 1 ^a^	Model 2 ^b^
		Beta^1^ (95% CI)	Beta^1^ (95% CI)
Females	Rarely lonely	Ref.	Ref.
	Lonely	4.32^**^ (1.65-6.10)	2.88 (-0.37-6.14)
Males	Rarely lonely	Ref.	Ref.
	Lonely	6.50^**^ (2.10-10.91)	5.61^*^ (0.54-10.67)

^a^ Adjusted for age (females *n*=1 186, males *n*=804), ^b^ Adjusted for age (females *n*=1 041, males *n*=741), socioeconomic status (SES), mental distress, close friends and self-rated health

^1^Unstandardized regression coefficient (B)

^*^ p < 0.05, ^**^ p < 0.01

In the sensitivity analyses related to abdominal obesity where participants with obesity at baseline were excluded, a positive linear relationship persisted in both models (Table S3). In females, the average WC was slightly lower than that reported in the main analysis, whereas in men, it was slightly greater (5.81 cm (95% CI: 0.59–11.04) than 5.61 (95% CI: 0.54–10.67), i.e., the relationship remained in males even with the reduced sample size. When we categorized loneliness into three levels, a positive relationship was still evident for those who were often lonely (Table S4). In this sensitivity analysis, both males and females showed higher average WC values compared to the main analysis; however, a statistically significant increase was observed only among females. Additionally, a positive linear relationship was observed for those who were sometimes lonely during adolescence.

## Discussion

This study is among the first to use repeated measures of loneliness and obesity from adolescence to adulthood. Our findings showed that adolescents who reported a high degree of loneliness had a greater risk of obesity as young adults than did those who rarely felt lonely. This risk remained present when adjusted for socioeconomic status, social network, mental health, and self-reported health. Both females and males had, on average, a higher BMI than young adults did if they reported being lonely as adolescents, and males also had, on average, a larger WC in young adulthood if they reported loneliness in adolescence. In general, the proportion of individuals with obesity increased from adolescence to young adulthood in both sexes. While the proportion of lonely females decreased, the level of loneliness was relatively stable in males. The proportion of adolescents who developed obesity between adolescence and adulthood was approximately twice as large for those who were lonely than for those who were rarely lonely. Despite the increased obesity rates from adolescence to young adulthood, loneliness rates either decreased (particularly among females) or remained stable (among males). This contrast suggests complex social dynamics, as the trends in loneliness and obesity do not directly align and may be influenced by various factors.

### Development of loneliness and obesity across adolescence and adulthood

In our sample from the Young-HUNT3 Survey (2006–08), the overall prevalence of loneliness among boys and girls was 8.9%. This percentage is higher than that previously reported in the Norwegian PISA survey 3–5 years earlier (7%) [37], and lower than that reported in the Ungdata survey a few years later (18%) [38]. The differences in prevalence rates can be explained by the general increase in reported loneliness among youths in Norway seen throughout the 2000 s [16, 39, 40]. More girls than boys reported loneliness, a trend that has been observed in all the Young-HUNT surveys [16] and the Norwegian Ungdata survey [40]. However, the disparity in loneliness levels between men and women tends to diminish as individuals progress into adulthood, as observed across multiple measurement intervals. A similar development of loneliness from adolescence to adulthood has been reported in previous studies [41, 42]. Consistent with our findings, several studies have shown minimal differences in loneliness between males and females during adulthood. However, research indicates that sex differences in loneliness may be more pronounced during certain life stages, such as adolescence [41, 43]. Hawkley and Capitanio emphasized that loneliness can impact health from an early age, with effects observable even in childhood (5–9 years of age) [17]. However, as individuals transition from adolescence to adulthood, a complex interplay of social and health factors emerges, revealing the “cultural loneliness paradox” [44]. Our findings suggest that sex differences in loneliness during specific life stages may stem from restrictive cultural norms that limit social autonomy or emotional expression, even within seemingly well-connected social networks. This supports the paradox’s premise: loneliness can persist in cultures where relationships are unfulfilling or constrained by social expectations. Interestingly, while obesity rates tend to rise during this life stage, loneliness does not follow the same pattern. Instead, it often decreases or remains stable. This divergence underscores the multifaceted nature of social connection and its distinct trajectory from other health outcomes.

The significant increase in overweight and obesity rates across our sample population aligns with research findings from Sweden and Germany during a comparable timeframe, suggesting a widespread increase in BMI across these regions [45], consistent with broader European observations of increasing obesity prevalence. Obesity was more common in boys than in girls in our study, which is consistent with research from other high-income countries [46]. In addition, there was a marked increase in abdominal obesity in both sexes.

Our results revealed approximately a threefold increase in obesity rates between the two data collection points. This is a larger increase than reported in studies from Australia and the United States [47, 48]. Our study also revealed that very few adolescents with obesity obtain normal weight into young adulthood, a trend that is in agreement with the findings in other studies. For individuals with grade 1 obesity (BMI 30.0-34.9), the annual probability of attaining normal body weight has been estimated to approximately 0.8% for women and 0.5% for men [49],, and the likelihood of attaining normal body weight decreases with increasing BMI categories. The increases in BMI and WC are a growing public health concern due to obesity-related health issues among Norwegian adolescents and young adults. The challenge of obtaining a healthier weight when one has developed overweight or obesity at a young age, underscores adolescence as a crucial period for preventive efforts of obesity.

Our results revealed that the proportion of young adults with obesity was highest among those who reported loneliness in adolescence (Fig. 2). The proportions of individuals with high BMIs and increasing BMI between adolescence and young adulthood were approximately twice as high among those who were lonely compared to those who were rarely lonely. These findings suggest that being lonely in adolescence may be one of the factors influencing unhealthy weight gain during the transition to adulthood, and hence of importance for preventative efforts of obesity.

Conversely, obesity can lead to social stigmatization, reduced self-esteem, and avoidance of social interactions (ref), which may further increase feelings of loneliness.

However, our sensitivity analyses, which excluded individuals with obesity at baseline, revealed a clear directional relationship between loneliness in adolescence and obesity in adulthood. This finding underscores the importance of developing targeted interventions to address the consequences of rising loneliness rates in adolescence, as these interventions may have significant implications for prevention of obesity.

### Prospective association between loneliness and obesity

Our research reveals a prospective link between adolescent loneliness and obesity in young adulthood. Qualter et al. also reported an association between loneliness and BMI via a similar methodology but with an indirect measure of loneliness (without explicitly using the term “lonely”) [50]. In their study, 12-year-old lonely girls had an increased BMI one year later, whereas lonely boys had a decreased BMI one year later. This finding contrasts with our results, which demonstrate a stronger association between adolescent loneliness and adult obesity in males than in females, with lonely males exhibiting higher odds of obesity and greater BMI increases from adolescence to adulthood.

This discrepancy may be attributed to differences in study design. Qualter et al. focused on a one-year period during the transition from childhood to adolescence, whereas our study encompassed a longer timeframe and examined an older cohort. Despite these variations, both studies confirmed that loneliness contributes to BMI increases over time.

Our study accounted for mental distress; a factor not adjusted for by Qualter et al. [50]. We were thus able to observe significant differences in mental distress between lonely individuals and rarely lonely individuals, prompting us to adjust for this variable because of established associations between depression and loneliness [23]and between depression and obesity [51]. The inclusion of consideration of additional variables (SES, close friends and self-rated health) the strength of associations slightly diminished remaining significant for both sexes.

Shiovitz-Ezra and Parag investigated the impact of loneliness on metabolic factors, including WC, and inflammatory markers in Americans aged 57–85 years [52]. While their research revealed a non-significant tendency toward increased WC risk among lonely individuals after a five-year period, our study demonstrated a statistically significant greater WC in lonely boys, specifically 5.61 cm greater (*p* < 0.05), over an eleven-year timeframe. Henriksen et al. examined the relationship between loneliness and metabolic syndrome, including elevated WC as part of the metabolic syndrome measure, using data from HUNT2 (1995-97) and HUNT3 (2006-08), with a mean participant age of 45 years [53]. Their results indicated that lonely individuals had a greater risk of metabolic syndrome ten years later, with a dose-response relationship between increased loneliness and metabolic syndrome risk. However, they did not analyze elevated WC risk separately. Although these studies encompass diverse age ranges, their findings seem primarily to disagree with each other. While our results support an association between loneliness, obesity, and abdominal obesity, it is important to interpret these findings considering differences in study populations, methodologies, and outcome measures across the literature. Our study contributes to the overall understanding of this relationship by utilizing a large, population-based cohort with prospective data and objective measures of obesity. However, variations in how loneliness and obesity are assessed, as well as differences in confounding factors, may account for inconsistencies in findings from previous research. Thus, our findings should be viewed as complementary to existing evidence, and future studies using harmonized measures and diverse samples are needed to further clarify these associations.

There are several pathways through which loneliness may affect the development of unhealthy weight gain and obesity in young adults. The relationship between adolescent loneliness and adult obesity could be mediated by factors such as disordered eating patterns as for instance binge eating and emotional eating, stress-related hormonal changes, or reduced physical activity, which are common coping mechanisms for loneliness [54]. Loneliness can lead to emotional distress, which some individuals may cope with by consuming high-calorie, fast foods. This behavior, known as emotional eating, can contribute to progressive weight gain and the development of obesity [55]. Loneliness has also been associated with negative eating behaviors, such as irregular meal patterns, increased snacking, and a preference for energy-dense, nutrient-poor foods [55]. These eating habits can lead to increased caloric intake and subsequent weight gain. The relationship between loneliness and adult WC could also be mediated by stress-induced cortisol production. Loneliness can activate the body’s stress response, leading to the release of cortisol and other stress hormones. Chronic stress and elevated cortisol levels can promote fat accumulation, particularly around the abdomen, which promotes abdominal fat deposition [56, 57] [58]. Moreover, individuals experiencing loneliness may be less likely to engage in physical activities, either due to a lack of motivation or social support [59] possibly contributing to weight gain and development of obesity [12].

These mechanisms highlight the complex interplay between psychological, behavioral, sociocultural and physiological factors in the relationship between loneliness and obesity. Understanding these pathways can influence development of targeted interventions to address both loneliness and obesity more effectively.

### Strengths and limitations

A main strength of this study is its large, population-based sample of adolescents drawn from Trøndelag County, Norway, and its longitudinal design. While the cohort is regional rather than strictly nationally representative, Trøndelag shares many demographic and socioeconomic characteristics with the broader Norwegian population, supporting the relevance of our findings. The long follow-up period further enabled a prospective examination of the association between loneliness in adolescence and obesity in young adulthood. This is an advantage compared to earlier studies, which have typically focused only on adolescents or adults separately with shorter follow-up periods. Furthermore, the analyses accounted for well-known confounders, including sex, age, psychological distress, physical activity, and socioeconomic status during adolescence. However, we cannot rule out the potential impact of residual confounders due to unknown or unmeasured factors, particularly when examining the potential longitudinal impacts of loneliness in adolescence.

There is a potential for selection bias, as only participants who attended both YH3 and HUNT4 were included. Previous analyses indicate that non-participants in the HUNT Study tend to have lower education levels and poorer health [60].

In addition to the anthropometric measurements, the included variables are based solely on self-reports, which can be prone to recall bias, especially concerning items assessing lifestyle behaviors. One must also consider that loneliness was measured with a single question. Direct questions require a greater understanding of the concept of loneliness and in the various response options (e.g., how often is “sometimes lonely”?). A single question generally has lower reliability and validity than the use of several questions does [61]. However, studies have shown that direct and indirect measures of loneliness have produced relatively similar results, with some indicating that men reported a lower degree of loneliness on direct questions [62]. Loneliness measured with a single question has also been shown to correlate well with larger scales [63], and a single question has the advantage that it produces fewer missing values ​​in the dataset than when several questions are used. Academic aspiration was used as an indicator of socioeconomic position (SES), reflecting certain aspects of students’ social and educational backgrounds. A key strength of this approach is its feasibility and relevance in large-scale surveys, where direct measures of SES may not always be available. Additionally, the use of a population-based Norwegian sample enhances the generalizability of the findings. However, the absence of additional SES, limits the overall comprehensiveness of the SES measure used in this study and may introduce bias or reduce comparability between subgroups.

## Conclusion

BMI and WC increase significantly from adolescence to adulthood, indicating a concerning trend toward higher rates of overweight, obesity, and abdominal obesity, which may lead to increased health risks. While overall loneliness levels have remained stable in the general population, a notable minority of young people consistently report experiencing a high degree of loneliness. Overall, this study suggests that loneliness in adolescence may be associated with an increased risk of BMI-defined obesity and higher WC in adulthood, with potentially stronger associations observed in males. Loneliness should be recognized as a risk factor for multiple chronic diseases and a public health concern, encouraging the enhancement of social connections as it may be a powerful strategy to prevent metabolic health issues. Awareness and screening of loneliness in adolescents might help identify those at risk for future development of obesity and related health problems. These points underscore the importance of a comprehensive approach to improving adolescent social and emotional well-being, given its potential long-term associations with physical and mental health outcomes.

## Supplementary Information

Additional file 1. Sensitivity analysis.

## Data Availability

Owing to restrictions imposed by the HUNT Research Centre, in accordance with the Norwegian Data Inspectorate’s guidelines, data cannot be made publicly available. The data are currently stored in the HUNT Databank, and restrictions exist for handling data files. The data may be available upon request to the HUNT Data Access Committee (hunt@medicine.ntnu.no). The HUNT data access information (available at http://www.ntnu.edu/hunt/data) describes in detail the policy regarding data availability.
