# Supplementary material for: Adolescent loneliness as a predictor of adult obesity: a longitudinal analysis from the HUNT study, Norway
Source: BMC Public Health. 2025 Aug 13;25:2751. doi: 10.1186/s12889-025-23872-0 (PMC12344875; doi:10.1186/s12889-025-23872-0)
Supplement: Supplementary file 1 — Additional file 1. Sensitivity analysis. [file 12889_2025_23872_MOESM1_ESM.docx]

**Additional file**

**Table of Contents**

**Table S1.** The association between loneliness in adolescence and BMI in adulthood: excluding obese participants at baseline.

**Table S2.** The association between loneliness in three categories in adolescence and BMI in adulthood.

**Table S3.** The association between loneliness in adolescence and waist circumference in adulthood: excluding obese Participants at baseline.

**Table S4.** The association between loneliness in three categories in adolescence and waist circumference in adulthood.

**Table S5.** The association between loneliness in adolescence and BMI in adulthood, pairwise deletion.

**Table S1** The association between loneliness in adolescence and BMI in adulthood: excluding obese participants at baseline

|  |  | **Model 1 ^a^** | | |  | **Model 2 ^b^** | | |
| --- | --- | --- | --- | --- | --- | --- | --- | --- |
|  |  | **OR overweight ^1^  (95% CI)** | **OR obesity ^1^**  **(95% CI)** | **B ^2^**  **(95% CI)** |  | **OR overweight ^1^ (95% CI)** | **OR obesity ^1^**  **(95% CI)** | **B ^2^**  **(95% CI)** |
| **F**emales | Rarely lonely | 1.0 | 1.0 | Ref. |  | 1.0 | 1.0 | Ref. |
|  | Lonely | 1.20  [0.75-1.92] | 2.18^**^  [1.31-3.63] | 1.60^**^  [0.65-2.55] |  | 1.17  [0.66-2.07] | 2.20^*^  [1.18-4.08] | 1.43^*^  [0.29-2.58] |
|  |  |  |  |  |  |  |  |  |
| **M**ales | Rarely lonely | 1.0 | 1.0 | Ref. |  | 1.0 | 1.0 | Ref. |
|  | Lonely | 0.92  [0.43-1.98] | 2.45^*^  [1.10-5.50] | 1.02  [-0.29-2.33] |  | 1.13  [0.47-2.71] | 2.39  [0.89-6.43] | 0.91  [-0.62-2.44] |
| ^a^ Adjusted for age (females n=1 084, males n=729), ^b^ Adjusted for age (females n=964, males n=676), SES, mental distress, close friends and self-rated health.  ^1^ Reference category: Normal weight/underweight  ^2^ Unstandardized regression coefficients (B)  ^*^ p < 0.05, ** p < 0.01 | | | | | | | | |

**Table S2** The association between loneliness in three categories in adolescence and BMI in adulthood

|  | | **Model 1 ^a^** | | |  | **Model 2 ^b^** | | |
| --- | --- | --- | --- | --- | --- | --- | --- | --- |
|  |  | **OR overweight ^1^  (95% CI)** | **OR obesity ^1^**  **(95% CI)** | **Beta ^2^**  **(95% CI)** |  | **OR overweight ^1^  (95% CI)** | **OR obesity ^1^**  **(95% CI)** | **Beta ^2^**  **(95% CI)** |
| **Females** | Rarely lonely | 1.0 | 1.0 | Ref. |  | 1.0 | 1.0 | Ref. |
|  | Sometimes lonely | 1.38^*^  (1.02-1.87) | 1.47^*^  (1.02-2.12) | 0.89^*^  (0.20-1.58) |  | 1.43^*^  (1.01-2.02) | 1.55^*^  (1.01-2.37) | 0.91^*^  (0.14-1.68) |
|  | Often lonely | 1.28  (0.82-2.00) | 2.32^**^  (1.45-3.71) | 1.89^**^  (0.91-2.87) |  | 1.39  (0.79-2.45) | 2.30^**^  (1.25-4.26) | 1.64^**^  (0.42-2.86) |
|  |  |  |  |  |  |  |  |  |
| **Males** | Rarely lonely | 1.0 | 1.0 | Ref. |  | 1.0 | 1.0 | Ref. |
|  | Sometimes lonely | 1.47  (0.99-2.19) | 1.57  (0.95-2.58) | 0.66  (-0.20-1.51) |  | 1.73^*^  (1.12-2.67) | 1.68  (0.97-2.89) | 0.79  (-0.12-1.71) |
|  | Often lonely | 1.14  (0.56-2.34) | 3.12^**^  (1.55-6.28) | 2.20^**^  (0.83-3.58) |  | 1.64  (0.72-3.74) | 3.15  (1.31-7.54) | 2.25^**^  (0.64-3.87) |
| ^a^ Adjusted for age (females n=1 204, males n=813), ^b^ Adjusted for age (females n=1 057, males n=749), SES, mental distress, close friends and self-rated health.  ^1^ Reference category: Normalweight/underweight  ^2^ Unstandardized regression coefficients (B)  ^*^ p < 0.05, ^**^ p < 0.01 | | | | | | | | |

**Table S3** The association between loneliness in adolescence and waist circumference in adulthood: excluding obese Participants at baseline

|  | | **Model 1 ^a^** | |  | **Model 2 ^b^** |
| --- | --- | --- | --- | --- | --- |
|  |  | **Beta^1^ (95% CI)** | |  | **Beta^1^ (95% CI)** |
| **Females** | Rarely lonely | | Ref. |  | Ref. |
|  | Lonely | | 1.84 (-1.02-4.70) |  | 2.05 (-1.26-5.36) |
|  |  | |  |  |  |
| **Males** | Rarely lonely | | Ref. |  | Ref. |
|  | Lonely | | 4.93^*^ (0.58-9.28) |  | 5.81^*^ (0.59-11.04) |
| ^a^ Adjusted for age (females n=720, males n=622), ^b^ Adjusted for age (females n=645, males n=579), SES, mental distress, close friends and self-rated health.  ^1^ Reference category: Normal weight/underweight  ^2^ Unstandardized regression coefficients (B)  ^*^ p < 0.05 | | | | | |

**Table S4** The association between loneliness in three categories in adolescence and waist circumference in adulthood

|  |  | **Model 1 ^a^** |  | **Model 2 ^b^** |
| --- | --- | --- | --- | --- |
|  |  | Beta^1^ (95% CI) |  | Beta^1^(95% CI) |
| **Females** | Rarely lonely | Ref. |  | Ref. |
|  | Sometimes lonely | 1.91 (-0.02-3.85) |  | 1.91 (-0.28-4.09) |
|  | Often lonely | 4.90^**^ (2.17-7.64) |  | 3.89^*^ (0.44-7.33) |
|  |  |  |  |  |
| **Males** | Rarely lonely | Ref. |  | Ref. |
|  | Sometimes lonely | 1.81 (-0.94-4.56) |  | 1.87 (-1.06-4.80) |
|  | Often lonely | 6.86^**^ (2.42-11.29) |  | 6.27^*^ (1.10-11.44) |
| ^a^ Adjusted for age (females n=1 186, males n=804), ^b^ Adjusted for age (females n=1 041, males n=741), SES, mental distress, close friends and self-rated health  ^1^ Reference category: Normal weight/underweight  ^2^ Unstandardized regression coefficients (B)  ^*^ p < 0.05, ^**^ p < 0.01^**^ p < 0.01 | | | | |

**Table S5** The association between loneliness in adolescence and BMI in adulthood, pairwise deletion (n=2053).

|  |  | **Model 1 ^a^** |  | **Model 2 ^b^** |
| --- | --- | --- | --- | --- |
|  |  | Beta^1^ (95% CI) |  | Beta^1^ (95% CI) |
| **Females** | Rarely lonely | Ref. |  | Ref. |
|  | Lonely | 1.62^**^ (0.66- 2.58) |  | 1.06 (-0.09-2.21) |
|  |  |  |  |  |
| **Males** | Rarely lonely | Ref. |  | Ref. |
|  | Lonely | 2.07^**^ (0.71-3.44) |  | 2.22^**^ (0.64-3.80) |
| ^a^ Adjusted for age (females n=1 204, males n=813), ^b^ Adjusted for age (females n=1 057, males n=749), SES, mental distress, close friends and self-rated health  ^1^ Reference category: Normal weight/underweight  ^2^ Unstandardized regression coefficients (B)  ^*^ p < 0.05, ^**^ p < 0.01 | | | | |
